# The mediating role of self‐esteem in the relationship between hope and life satisfaction among university students during a global health crisis

**DOI:** 10.1002/hsr2.2311

**Published:** 2024-08-12

**Authors:** Stephen Cheong Yu Chan, Qi Lu Huang

**Affiliations:** ^1^ Felizberta Lo Padilla Tong School of Social Sciences Saint Francis University Tseung Kwan O Hong Kong; ^2^ Department of Social and Behavioural Sciences City University of Hong Kong Kowloon Hong Kong

**Keywords:** COVID‐19, hope, life satisfaction, self‐esteem, university students

## Abstract

**Background and Aims:**

The worldwide health emergency sparked by the COVID‐19 pandemic has deeply shaken educational environments, posing unprecedented challenges to university students’ well‐being. While individual links between self‐esteem, hope, and well‐being are established, their combined impacts during crises remain underexplored. Our study addresses this gap by investigating the interplay among hope, self‐esteem, and life satisfaction within the context of university students navigating the challenges posed by the pandemic.

**Methods:**

Conducting an online cross‐sectional self‐administered survey during Hong Kong's third pandemic wave, we measured hope, self‐esteem, and life satisfaction using validated scales. Three hundred and fifteen university students (211 females; mean age = 22.08; *SD* = 2.74) participated.

**Results:**

Through multiple regression and mediation analyses, our findings indicate that university students with elevated hope and self‐esteem scored higher on life satisfaction measures. Specifically, our analysis revealed that self‐esteem may serve as a partial mediator in the relationship between hope and life satisfaction, highlighting how heightened hope indirectly enhances life satisfaction by strengthening self‐esteem.

**Conclusion:**

This study reveals intricate relationships among hope, self‐esteem, and life satisfaction in university students, particularly during external adversities like the COVID‐19 pandemic. The implications extend to mental health interventions, emphasizing the potential benefits of cultivating hope and self‐esteem to enhance life satisfaction among university students confronting formidable challenges.

## 1 INTRODUCTION

The global outbreak of COVID‐19 pandemic brought about substantial shifts in education, including the implementation of social distancing measures and the transition to online learning.^1^ This sudden change from traditional in‐person classrooms to virtual platforms presented unique challenges for different populations, including young adults, educators, and families.^2^ For university students navigating this transition, the shift disrupted established routines, limited social interactions, and heightened reliance on self‐regulated learning, presenting considerable challenges.^3^ Consequently, this shift has resulted in not only academic obstacles but also potential harm to their mental well‐being,^4^ posing a greater risk for mental health issues.^5^

Quarantine measures, in particular, have induced profound effects. Apart from fostering physical isolation, these restrictions have also imposed psychological seclusion.^6^ Prolonged periods of quarantine can result in adverse psychological effects, including the manifestation of symptoms associated with posttraumatic stress, feelings of confusion, and increased levels of anger. In addition, people who have undergone quarantine are more likely to experience exhaustion, a feeling of disconnection from others, anxiety, irritability, sleep disturbances, diminished focus, and indecisiveness, which can further deteriorate their overall psychological well‐being.^7^

Hong Kong is no exception, the implementation of societal lockdowns and strict quarantine measures has presented significant mental health challenges across various student demographics, including primary school children^8^ and emerging adults in university.^2^ The sudden suspension of face‐to‐face classes and the hasty shift to remote learning modalities caused by the pandemic has particularly impacted the quality of interactions among teachers and students, as well as among students themselves, affecting cognitive engagement and the process of knowledge acquisition and application.^2^ This situation is likely exacerbated for university students due to heightened academic expectations, pressures, and the demand for self‐regulated learning.^9^

These findings underscore the influences of global adversity on young adult undergraduates’ learning and mental health conditions. To counteract these negative impacts and safeguard well‐being during such crises, it is essential to identify protective factors like hope and self‐esteem.

### 1.1 Hope and self‐esteem as potential protective factors in young adults during adversity

Hope is conceptualized as a positive cognitive state represented by the formulation of plans and determination to achieve goals despite uncertainty regarding their success.^10^ Specifically, Snyder et al.^11^ define hope as a cognitive construct involving two distinctive yet interrelated components: agency thinking (directed energy) and pathways thinking (plans to achieve goals). Both components are associated with subjective well‐being, suggesting that hopeful young adults may maintain a stronger sense of fulfillment, even during challenging times, which enhances their positive sense of subjective well‐being.

Similarly, self‐esteem, the evaluative aspect of self‐concept,^12^ plays a pivotal role in psychological well‐being. It can be further differentiated at the individual, collective, and relational levels. Regardless of the level, young adults with higher self‐esteem appeared to have better subjective well‐being.^13^, ^14^ These findings suggest that interventions aimed at promoting self‐esteem and hope may serve as effective resilience‐building strategies against psychological challenges during difficult periods.

Internationally, there have been some studies providing evidence of the importance of hope and self‐esteem during the COVID‐19 pandemic. For instance, Flesia et al.^15^ explored the role of hope on psychological distress related to COVID‐19 in Pakistan, they found that higher levels of hope were associated with reduced levels of psychological distress. Azmi et al.^16^ found that university students with high self‐esteem develop lower chances of getting depressive symptoms by 17% during the COVID‐19 pandemic. While these studies are exploring the detrimental effects of the COVID‐19 pandemic on people's mental well‐being and happiness,^4^, ^17^ there is limited exploration into how protective factors might bolster life satisfaction, particularly among Asian young adults in university settings. Moreover, the combined influence of these factors during a large‐scale crisis (e.g., the COVID‐19 pandemic), warrants further investigation.

### 1.2 The protective role of hope and self‐esteem: Insights from the Broaden‐and‐Build theory

Barbara Fredrickson's Broaden‐and‐Build Theory^18^ provides a compelling framework for understanding the role of positive emotions, such as hope, in nurturing psychological resilience and well‐being among young adults. It is theorized that such emotions expand one's momentary thought‐action repertoire, fostering the development of enduring personal resources, spanning physical, intellectual, social, and psychological domains. These resources function as a safeguard against the detrimental effects of adversity, bolstering young adults’ capacity to handle stressful situations with greater ease.

In line with this theory, hope, as one of the positive emotions, plays a significant role in coping with adversities, including the unprecedented stressors precipitated by the COVID‐19 pandemic. Previous research suggests that hope is a protective factor against the development of psychopathology during stressful situations^19^ and enhances subjective well‐being by promoting a sense of control and efficacy.^20^ Furthermore, hope, specifically pathways thinking—defined as an individual's perceived ability to discern pathways toward desired goals—has been recognized as a mediator of depressive symptoms in young adults who experience negative emotions.^21^ This echoes Fredrickson's assertion that hope can counteract the propensity of distressing events to constrict an individual's perspective and induce feelings of depression. Instead, hope can promote cognitive flexibility and innovative thinking. This, in turn, equips young adults with the capacity to formulate effective problem‐solving strategies and maintain a more positive outlook, even when confronted with daunting challenges.^22^

Self‐esteem, or positive self‐evaluations, may also play a crucial role in constructing psychological resilience. It shapes a positive self‐concept, serving as a protective factor against mental health issues.^23^ According to Orth et al.,^24^ people with higher self‐esteem are less likely to experience stress and tend to use more effective coping strategies when faced with challenging situations. This supports the argument of the Broaden‐and‐Build Theory,^18^ which suggests that positive emotions and thoughts, such as self‐esteem, can broaden an individual's perspective and enhance their resilience. The Adolescent Resilience Model^25^ has also demonstrated that self‐esteem can serve as a buffer and reduce the negative effects of life's hardships. Based on the existing literature, it appears that maintaining good levels of self‐esteem and hope can be vital in promoting the well‐being of young adult undergraduates, particularly in unfavorable circumstances.

The previous section has indicated that self‐esteem and hope could be inter‐related. However, as proposed by Snyder's Hope Theory,^26^ people will focus on the goal‐pursuit process, when they can achieve the goals, emotions and esteem could be developed. For example, Cheavens et al.^27^ revealed that hope therapy could promote life meaning and self‐esteem as well as reduce depressive and anxiety symptoms.

### 1.3 Current study

Despite the increasing amount of research highlighting the possible influence of hope and self‐esteem on psychological well‐being, there is still limited knowledge regarding the interplay of these factors, especially in terms of life satisfaction among young adults. This gap is especially voiced in the context of external adversities such as the COVID‐19 pandemic. Gaining a better understanding of the interactions between these factors could yield significant insights for enhancing young adult undergraduates’ well‐being during challenging times.

In response to this gap in the literature, the present study sought to investigate the association between hope, self‐esteem, and life satisfaction among a sample of young adult university students. Drawing upon the theoretical frameworks of the Broaden‐and‐Build Theory, Hope Theory, and the Adolescent Resilience Model, we hypothesized a positive relationship between life satisfaction, hope, and self‐esteem. Further, we posited that self‐esteem may act as a mediator in the link between hope and life satisfaction, thereby providing a deeper understanding of the intricate interplay among these variables and their impact on the mental well‐being of young adults.

## 2 METHODS

### 2.1 Study design

This study adopted a cross‐sectional design and utilized an online self‐administered questionnaire for data collection during Hong Kong's third pandemic wave (February to July 2021).

### 2.2 Participants and procedures

Participants in this study were recruited using an opportunity sampling approach in Hong Kong. A total of 315 Chinese young adult undergraduates, with a mean age of 22.08 years (*SD* = 2.74), constituted the final sample. The human research ethics committee at the university granted ethical approval for this study, ensuring its compliance with the established ethical guidelines and protocols.

Recruitment was conducted through the university's mass e‐mail system, with over 500 e‐mails sent to potential participants. As we sent invitations via the internal email system, the inclusion criteria were full‐time university students, while the exclusion criteria were those who were not studying at the university and those who had difficulties in reading and comprehension. Of those contacted, 315 individuals completed the questionnaires. Before participation, all participants acquired informed consent online, emphasizing the voluntary nature of their commitment. The survey was administered using the Qualtrics platform, the participants could withdraw at any stage if they felt uncomfortable.

To ensure the integrity and confidentiality of the data, young adult undergraduates were requested to provide personal identifiers, such as their study major and student number. However, these identifiers were subsequently removed during the data analysis phase to maintain data anonymization.

### 2.3 Measures

The study questionnaire involved three validated scales in measuring key psychological attributes. Apart from these, data regarding personal and socio‐demographic details were collected. These details included age, sex, personal monthly income, and expenditure. Personal monthly income was categorized into two brackets for analysis: “1” represented a monthly income ranging from “HK$0 to HK$5,999,” and “2” denoted a monthly income of “more than HK$6,000.” A 5‐point Likert scale was used to evaluate financial satisfaction (i.e., perceived sufficiency of personal expenditure), with 1 representing “very insufficient” and 5 suggesting “very sufficient.” This measure aimed to capture participants’ subjective assessment of their financial adequacy in relation to their expenditures.^28^, ^29^

The Dispositional Hope Scale (DHS)^30^ was employed to gauge the level of hope in this study. The scale consisted of 12 items, including four filler items. The total score was obtained by summing the eight remaining items, which ranged from 8 to 64, and calculating the average. Higher scores indicated more significant levels of hope. According to this study, the DHS is highly reliable (Cronbach's alpha = 0.90).

Regarding self‐esteem, the Rosenberg Self‐Esteem Scale (RSE) was employed in this study. The scale consisted of 10 items developed by Rosenberg.^31^ The total scores, ranging from 1 to 4, were computed by averaging the responses, with higher scores reflecting greater levels of self‐esteem. The RSE used in our study was reliable (Cronbach's alpha = 0.86).

The participants’ life satisfaction was assessed using Diener et al.'s^32^ Satisfaction with Life Scale (SWLS). The SWLS is comprised of a set of five items designed to measure individuals’ overall satisfaction with their lives. Scores on the SWLS range from 7 to 35, with higher scores indicating higher levels of life satisfaction. In this study, the average scores on the SWLS were analyzed, and the scale demonstrated good reliability (Cronbach's alpha = 0.86).

### 2.4 Data analyses

The SPSS version 25.0 was utilized for data analysis. A descriptive analysis was conducted, followed by correlational analyses to explore the relationship between different variables. After that, a multiple regression analysis was performed to test the influence of self‐esteem on the association between hope and life satisfaction. For this purpose, the Hayes^33^ PROCESS Macro ‐ Model 4 was utilized, with hope being the exogenous variable, self‐esteem as the mediator, and SWLS as the endogenous variable. Finally, to examine the indirect effects, a bias‐corrected bootstrapping method was employed, which involved generating 10,000 bootstrap samples and computing each effect's 95% confidence interval to account for possible biases.

## 3 RESULTS

### 3.1 Descriptive statistics

Our study encompassed 315 young adults, with an average age of 22.08 years and a standard deviation of 2.74. About two‐thirds of these individuals were female. The bulk of participants (77.1%) reported monthly earnings below HK$6000 (approximately US$750), yet they remained optimistic about their financial management capabilities (Mean = 3.16, *SD* = 0.99). For a detailed analysis of our sample's characteristics, please see Table 1.

**Table 1:** Descriptive statistics.

	Overall samples (*N* = 315)
	Frequency (%)/Mean (*SD*)
Variables	
Age (years)	22.08 (2.74)
Sex	
Male	104 (33%)
Female	211 (67%)
Major Disciplines^a^	
Creative Arts	69 (25%)
Humanities and Language	71 (26%)
Social Sciences	138 (49%)
Year Standing^a^	
Year 1	45 (16%)
Year 2	76 (27%)
Year 3	69 (25%)
Year 4	88 (32%)
Monthly Income (HK$)	
<$6000	243
$6000 or above	72
Financial Satisfaction	3.16 (0.99)
Hope	5.01 (1.28)
Self‐esteem	2.48 (0.48)
Life Satisfaction	3.87 (1.20)

^a^ Number of valid responses was 278.

### 3.2 Correlational analyses

Table 2 provides a summary of the correlational analyses conducted between life satisfaction, as measured by the SWLS, and selected variables under study. Within the socio‐demographic category, the SWLS demonstrated a significantly positive correlation with expenditure (*r* = 0.31, *p* < 0.001). Additionally, the study uncovered positive and statistically significant correlations between SWLS and the psychological constructs of hope and self‐esteem (*ps* < 0.001), highlighting strong links between these factors and overall life satisfaction.

**Table 2:** Correction matrix among all measures with Satisfaction with Life Scale (SWLS).

	Sex	Income	Financial satisfaction	Hope	Self‐esteem	SWLS
Age	−0.08	0.40***	−0.15**	0.10	0.09	0.02
Sex		−0.18**	0.15**	−0.05	−0.01	0.03
Income			0.02	0.12*	0.09	0.00
Financial Satisfaction				0.17**	0.20***	0.31***
Hope					0.55***	0.58***
Self‐esteem						0.52***

*Note*: SWLS = Life Satisfaction.

* *p* < 0.05

** *p* < 0.01

*** *p* < 0.001.

### 3.3 Multiple regression analyses

The regression analysis results, presented in Table 3, indicated that expenditure (*β* = 0.23, *p* < 0.001), hope (*β* = 0.39, *p* < 0.001), and self‐esteem (*β* = 0.67, *p* < 0.001) were significantly and positively associated with life satisfaction. These three factors together accounted for 43.3% of the variance in life satisfaction scores. Given the significance of financial satisfaction, we included it as a covariate in the following mediation analysis.

**Table 3:** Multiple linear regression results for testing hope and self‐esteem in relation to life satisfaction.

Variables	*β* value	Standard error	Standardized *β* value	*t* value	*p* value
Age	0.01	0.02	0.01	0.28	0.778
Sex	0.04	0.11	0.02	0.37	0.714
Income	−0.22	0.14	−0.08	−1.61	0.108
Financial Satisfaction	0.23	0.06	0.19	4.17	<0.001
Hope	0.39	0.05	0.41	7.93	<0.001
Self‐esteem	0.67	0.13	0.27	5.10	<0.001

### 3.4 Mediation analyses

Upon controlling for covariates, a mediation analysis was conducted to explore the extent to which self‐esteem mediates the association between hope and life satisfaction among Chinese young adults. The overall regression model assessing the influence of hope on life satisfaction yielded significant results [*F*(2, 312) = 95.57, *p* < 0.001], demonstrating a robust effect size (*f* ^2^ = 0.35).^34^

**Figure 1:**
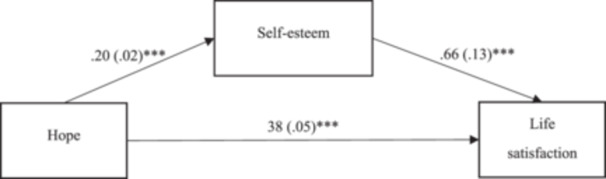
Results of analysis testing for self‐esteem as a mediator in the association between hope and life satisfaction in Chinese undergraduates. All numbers represent non‐standardized regression coefficients and their standard errors. *N* = 315. *p < 0.05, **p < 0.01, ***p < 0.001.

As depicted in Figure 1, the empirical findings indicate that hope exerts a direct impact on both self‐esteem (*β* = 0.20, *p* < 0.001) and life satisfaction (*β* = 0.38, *p* < 0.001). Furthermore, a positive association between self‐esteem and life satisfaction (*β* = 0.66, *p* < 0.001) was observed.

To be more specific, the analysis uncovered a significant indirect effect of hope on life satisfaction mediated through self‐esteem (*c*’ = 0.13, *p* < 0.001). Given the substantial direct effect of hope on life satisfaction, it is posited that self‐esteem serves as a partial mediator in the nexus between hope and life satisfaction. This mediating dynamic is elaborately illustrated in Figure 1.

## 4 DISCUSSION

In the face of the ongoing global crisis precipitated by COVID‐19, our study offers invaluable insights into the mental health of young adult university students, a population particularly susceptible to the psychosocial effects of the pandemic. This study contributes to the existing literature by investigating the interplay among the variables of interest, specifically highlighting the mediating mechanism of self‐esteem in connecting hope and life satisfaction among Chinese young adult undergraduates who are confronted with difficult circumstances.

In line with existing literature on the mental well‐being of young adults, our research conducted with Chinese university students demonstrates a moderate positive association between hope, self‐esteem, and life satisfaction.^35^ Our findings indicate that higher‐hope individuals appear to view situations more positively, which may impact their overall well‐being.^36^ Simultaneously, those with elevated self‐esteem may perceive themselves more favorably, a perspective that could manifest in heightened life satisfaction scores. These results underscore the significant role of these attributes in mitigating adverse mental health outcomes.

Furthermore, our multiple regression analysis underscores the substantial influence of self‐esteem on protecting young people's life satisfaction, especially during the time of global health crisis. These results reinforce previous research that identifies self‐esteem as a crucial predictor of subjective well‐being for this group.^37^

Of particular importance, our study identifies self‐esteem as a key factor that partially mediates the relationship between hope and life satisfaction, underscoring its pivotal role in bolstering young adults’ well‐being during pandemics. In essence, hopeful undergraduate students possess the capacity to chart pathways and muster determination to pursue goals even amidst adverse circumstances. Drawing upon the Broaden‐and‐Build Theory, the hopeful outlook of young adults may not only foster a sense of well‐being but also broaden their scope of attention, prompting them to identify and pursue positive life opportunities.^18^ This optimistic mindset may further cultivate the perception of having sufficient resources, subsequently enhancing life satisfaction. In contrast, individuals with low self‐esteem may be more susceptible to negative self‐evaluations, especially when faced with critical feedback.^38^ Additionally, our study supports Haase's^25^ Adolescent Resilience Model, highlighting the role of high self‐esteem in facilitating social adaptation among students and serving as a protective factor against the impact of negative feedback on their self‐esteem.

In short, our study illustrates the reinforcing dynamic among hope, self‐esteem, and life satisfaction in young adults. On one hand, we discovered that young adults who possess a high degree of hope often feel positive emotions and improve their life satisfaction via positive reactions to their surroundings. Conversely, those with elevated life satisfaction demonstrate greater motivation for goal attainment and tend to harbor more favorable self‐appraisals. This reciprocal relationship creates a virtuous cycle, wherein hope and life satisfaction mutually reinforce each other through the mechanism of self‐esteem.

### 4.1 Implications

Understanding the interplay between hope, self‐esteem, and life satisfaction offers an evidence‐based framework for mental health and educational practitioners to support young adult university students, particularly during stressful circumstances such as a pandemic. Enhancing hope and self‐esteem should not be peripheral endeavors but the central strategies for promoting resilience and overall well‐being in this demographic. Practical examples include integrating hope and self‐compassion into curricula, establishing realistic goal‐setting workshops, fostering optimistic thinking through student mentorship, and reinforcing positive self‐affirmations as part of daily practice. These strategies, tailored to young adults’ unique demands, could form comprehensive mental well‐being programs within higher education institutions.

Our findings underscore the significant mediating role of self‐esteem in the relationship between hope and life satisfaction, suggesting that interventions aimed at increasing self‐esteem could amplify the positive effects of hope on life satisfaction. This is supported by existing literature, such as Orth et al.,^39^ who demonstrated that self‐esteem significantly predicts future subjective well‐being and life satisfaction. Furthermore, interventions focusing on enhancing self‐esteem have shown promising results in improving mental health outcomes among university students.^40^ These insights are invaluable for designing interventions that target both hope and self‐esteem to maximize their impact on life satisfaction.

The potential benefits are far‐reaching, enabling young adults to manage stress effectively, enhance life satisfaction, and build resilience necessary for overcoming current and future adversities. By weaving these insights into the fabric of higher education, we empower young adults not only to survive challenging times but also to flourish with skills and attitudes that will serve them well beyond the crisis. This dual focus on hope and self‐esteem can help mitigate the adverse effects of crises like the COVID‐19 pandemic and promote a more robust sense of well‐being among this vulnerable population.

### 4.2 Limitations and future directions

Despite its contributions, it is essential to acknowledge some limitations of our study. First, our sample was drawn from one university, which may potentially limit the generalizability of findings. Second, due to the COVID‐19 pandemic and the urgency to understand its impact on respondents, we were unable to conduct a power analysis to determine an ideal sample size. We aimed to recruit as many participants as possible, but a larger sample size was not guaranteed. Third, our participants were all Chinese university students. Previous research suggests that the roles of hope and self‐esteem may vary among different racial groups.^41^ Therefore, cultural influences may not be fully accounted for.

Future research should aim to include a more diverse set of cultural and ethnic groups and involve multiple universities to enhance representativeness. Understanding the generalized and cross‐cultural interplay of hope, self‐esteem, and life satisfaction could offer invaluable insights into young adults’ resilience amidst global crises like the COVID‐19 pandemic. By addressing these challenges, we can develop robust and effective mental health strategies that safeguard and enhance young adults’ mental well‐being.

## 5 CONCLUSION

This investigation reveals the critical intermediary role that self‐esteem serves in the relationship between hope and life satisfaction, with an emphasis on Chinese university students during the pandemic and the shifting dynamics of education. This study underscores the necessity for the development and implementation of educational and mental health interventions aimed at promoting hope and self‐esteem. Such initiatives are essential for enhancing students’ resilience, improving their ability to cope, and increasing their overall satisfaction with life. Future research is urged to explore these intricate relationships in diverse cultural and socioeconomic contexts, providing further insights to inform comprehensive mental health interventions. Investing in these strategies will not only assist young adults in navigating current adversities but also equip them to thrive in a post‐pandemic world.

## AUTHOR CONTRIBUTIONS

**Stephen Cheong Yu Chan:** Conceptualization; data curation; investigation; methodology; project administration; formal analysis; writing—original draft; writing—review & editing. **Qi Lu Huang:** Conceptualization; data curation; investigation; methodology; project administration; formal analysis; writing—original draft; writing—review & editing. All authors have read and approved the final version of the manuscript. Qi Lu Huang and Stephen Cheong Yu Chan had full access to all of the data in this study and takes complete responsibility for the integrity of the data and the accuracy of the data analysis.

## ETHICS STATEMENT

All procedures performed in studies involving human participants were in accordance with the ethical standards of the institutional and/or national research committee and with the 1964 Helsinki Declaration and its later amendments or comparable ethical standards. The study was approved by the Research Ethics Committee of Hong Kong Metropolitan University (REC Ref. no.: HE‐SF2021/02). Informed consent was obtained from all individuals participating in the study. All participants provided written informed consent for the use of anonymized data.

## TRANSPARENCY STATEMENT

The lead author Qi Lu Huang affirms that this manuscript is an honest, accurate, and transparent account of the study being reported; that no important aspects of the study have been omitted; and that any discrepancies from the study as planned (and, if relevant, registered) have been explained.

## Data Availability

The data that support the findings of this study are available on request from the corresponding author. The data are not publicly available due to privacy or ethical restrictions.

## References

[1] Wang G , Zhang Y , Zhao J , Zhang J , Jiang F . Mitigate the effects of home confinement on children during the COVID‐19 outbreak. The Lancet. 2020;395(10228):945‐947. 10.1016/S0140-6736(20)30547-X PMC7124694 32145186

[2] Law VTS , Yee HHL , Ng TKC , Fong BYF . Transition from traditional to online learning in Hong Kong tertiary educational institutions during COVID‐19 pandemic. Technol Knowledge Learn. 2022;28:1425‐1441. 10.1007/s10758-022-09603-z

[3] Bozkurt A , Sharma RC . Emergency remote teaching in a time of global crisis due to CoronaVirus pandemic. Asian J Dist Educ. 2020;15(1):i‐vi. 10.5281/zenodo.3778083

[4] Henseke G , Green F , Schoon I . Living with COVID‐19: subjective well‐being in the second phase of the pandemic. J Youth Adolesc. 2022;51(9):1679‐1692. 10.1007/s10964-022-01648-8 35788856 PMC9252564

[5] Charles NE , Strong SJ , Burns LC , Bullerjahn MR , Serafine KM . Increased mood disorder symptoms, perceived stress, and alcohol use among college students during the COVID‐19 pandemic. Psychiatry Res. 2021;296:113706. 10.1016/j.psychres.2021.113706 33482422 PMC7781902

[6] Brooks SK , Webster RK , Smith LE , et al. The psychological impact of quarantine and how to reduce it: rapid review of the evidence. The Lancet. 2020;395(10227):912‐920. 10.1016/S0140-6736(20)30460-8 PMC7158942 32112714

[7] Hawryluck L , Gold WL , Robinson S , Pogorski S , Galea S , Styra R . SARS control and psychological effects of quarantine, Toronto, Canada. Emerging Infect Dis. 2004;10(7):1206‐1212. 10.3201/eid1007.030703 PMC3323345 15324539

[8] Ye FT‐F , Gao X , Sin K‐F , Yang L . Remote learning and mental health during the societal lockdown: a study of primary school students and parents in times of COVID‐19. BMC Public Health. 2023;23(1):1106. 10.1186/s12889-023-16040-9 37286984 PMC10246532

[9] Zhai Y , Du X . Addressing collegiate mental health amid COVID‐19 pandemic. Psychiatry Res. 2020;288:113003. 10.1016/j.psychres.2020.113003 32315885 PMC7162776

[10] Martin AM . Hopes and dreams. Philos Phenomenol Res. 2011;83(1):148‐173. 10.1111/j.1933-1592.2010.00422.x

[11] Snyder CR , Shorey HS , Cheavens J , Pulvers KM , Adams III VH , Wiklund C . Hope and academic success in college. J Educ Psychol. 2002;94:820‐826. 10.1037/0022-0663.94.4.820

[12] Neiss MB , Sedikides C , Stevenson J . Self‐esteem: a behavioural genetic perspective. Eur J Personality. 2002;16(5):351‐367. 10.1002/per.456

[13] Crocker J , Luhtanen R , Blaine B , Broadnax S . Collective self‐esteem and psychological Well‐Being among White, Black, and Asian College Students. Pers Soc Psychol Bull. 1994;20(5):503‐513. 10.1177/01461672942050

[14] Du H , King RB , Chi P . Self‐esteem and subjective well‐being revisited: the roles of personal, relational, and collective self‐esteem. PLoS One. 2017;12(8):e0183958. 10.1371/journal.pone.0183958 28841716 PMC5571946

[15] Flesia L , Adeeb M , Waseem A , Helmy M , Monaro M . Psychological distress related to the COVID‐19 pandemic: the protective role of hope. Eur J Invest Health Psychol Educ. 2023;13(1):67‐80. 10.3390/ejihpe13010005 PMC9857999 36661755

[16] Azmi FM , Khan HN , Azmi AM , Yaswi A , Jakovljevic M . Prevalence of COVID‐19 pandemic, self‐esteem and its effect on depression among university students in Saudi Arabia. Front Public Health. 2022;Feb 8 10:836688. 10.3389/fpubh.2022.836688 35211449 PMC8863063

[17] Luthra S , Agrawal S , Kumar A , Sharma M , Joshi S , Kumar J . Psychological well‐being of young adults during COVID‐19 pandemic: lesson learned and future research agenda. Heliyon. 2023;9(5):e15841. 10.1016/j.heliyon.2023.e15841 37159682 PMC10156410

[18] Fredrickson BL . The role of positive emotions in positive psychology: the broaden‐and‐build theory of positive emotions. Am Psychol. 2001;56:218‐226. 10.1037/0003-066X.56.3.218 11315248 PMC3122271

[19] Arnau RC , Rosen DH , Finch JF , Rhudy JL , Fortunato VJ . Longitudinal effects of hope on depression and anxiety: a latent variable analysis. J Pers. 2007;75(1):43‐64. 10.1111/j.1467-6494.2006.00432.x 17214591

[20] Chang EC , Chang OD , Kamble SV . Examining the relationship between positive mood and life satisfaction in Easterners and Westerners: is feeling good associated with building agency, broadening pathways, or both? J Happiness Stud. 2019;20(7):2159‐2172. 10.1007/s10902-018-0043-7

[21] Chan SCY , Huang QL . “Better hope, less depressed”: the potential mediating role of pathways thinking between negative emotions and depressive symptoms among Chinese university students. Hong Kong J Soc Work. 2022;56(01n02):3‐19. 10.1177/00914150231207999

[22] Fredrickson BL , Branigan C . Positive emotions broaden the scope of attention and thought‐action repertoires. Cogn Emot. 2005;19(3):313‐332. 10.1080/02699930441000238 21852891 PMC3156609

[23] Orth U , Robins RW . Understanding the link between low self‐esteem and depression. Curr Direct Psychol Sci. 2013;22:455‐460. 10.1177/0963721413492763

[24] Orth U , Robins RW , Meier LL . Disentangling the effects of low self‐esteem and stressful events on depression: findings from three longitudinal studies. J Pers Soc Psychol. 2009;97(2):307‐321. 10.1037/a0015645 19634977

[25] Haase JE . The adolescent resilience model as a guide to interventions. J Pediatr Oncol Nurs. 2004;21(5):289‐299. 10.1177/1043454204267922 15381798

[26] Snyder CR . TARGET ARTICLE: hope theory: rainbows in the mind. Psychol Inq. 2002;13(4):249‐275. 10.1207/S15327965PLI1304_01

[27] Cheavens JS , Feldman DB , Gum A , Michael ST , Snyder CR . Hope therapy in a community sample: a pilot investigation. Soc Indic Res. 2006;77:61‐78. 10.1007/s11205-005-5553-0

[28] Garrett S , James III RN . Financial ratios and perceived household financial satisfaction. J Finan Ther. 2013;4(1):4. 10.4148/jft.v4i1.1839

[29] Hira TK , Mugenda OM . Predictors of financial satisfaction: differences between retirees and non‐retirees. J Finan Counsel Plan. 1998;9(2):75‐84.

[30] Snyder CR , Irving LM , Anderson JR . Hope and health. In: Snyder CR , Forsyth DR , eds. Handbook of social and clinical psychology: the health perspective. Pergamon Press; 1991:285‐305.

[31] Rosenberg M . Society and the adolescent self‐image. Princeton University Press; 2015. 10.1515/9781400876136

[32] Diener E , Emmons RA , Larsen RJ , Griffin S . The satisfaction with life scale. J Pers Assess. 1985;49(1):71‐75. 16367493 10.1207/s15327752jpa4901_13

[33] Hayes AF . Introduction to mediation, moderation, and conditional process analysis: a regression‐based approach. Guilford Press; 2013.

[34] Cohen J , Cohen P , West SG , Aiken LS . Applied multiple regression/correlation analysis for the behavioral sciences. 3rd ed. Routledge; 2013. 10.4324/9780203774441

[35] Rand KL , Shanahan ML , Fischer IC , Fortney SK . Hope and optimism as predictors of academic performance and subjective well‐being in college students. Learn Individual Diff. 2020;81:101906. 10.1016/j.lindif.2020.101906

[36] Díaz D , Stavraki M , Blanco A , Gandarillas B . The eudaimonic component of satisfaction with life and psychological well‐being in Spanish cultures. Psicothema. 2015;27(3):247‐253. 10.7334/psicothema2015.5 26260932

[37] Diener E , Diener M . Cross‐cultural correlates of life satisfaction and self‐esteem. J Pers Soc Psychol. 1995;68(4):653‐663. 10.1037/0022-3514.68.4.653 7738768

[38] Brown JD , Marshall MA . The three faces of self‐esteem. Self‐esteem issues and answers: a sourcebook of current perspectives. Psychology Press; 2006:4‐9.

[39] Orth U , Robins RW . The development of self‐esteem. Curr Direct Psychol Sci. 2014;23(5):381‐387. 10.1177/0963721414547414

[40] Bruhns A , Lüdtke T , Moritz S , Bücker L . A mobile‐based intervention to increase self‐esteem in students with depressive symptoms: randomized controlled trial. JMIR Mhealth Uhealth. 2021;9(7):e26498. 10.2196/26498 34255711 PMC8314153

[41] Chang EC , Chang OD , Li M , et al. Positive emotions, hope, and life satisfaction in Chinese adults: a test of the broaden‐and‐build model in accounting for subjective well‐being in Chinese college students. J Positive Psychol. 2019;14(6):829‐835. 10.1080/17439760.2019.1579358

